# Isolation and characterization of a *Chlamydia muridarum tc0237* mutant from a genetic screen that is attenuated in epithelial cells

**DOI:** 10.1371/journal.pone.0329637

**Published:** 2025-08-05

**Authors:** Kaylee R. Jacobs, Caleb M. Ardizzone, Arkaprabha Banerjee, Evelyn Toh, Xiaoli Zhang, David E. Nelson

**Affiliations:** 1 Department of Microbiology and Immunology, Indiana University School of Medicine, Indianapolis, Indiana, United States of America; 2 Department of Microbiology, Perelman School of Medicine, University of Pennsylvania, Philadelphia, Pennsylvania, United States of America; UTHSCSA: The University of Texas Health Science Center at San Antonio, UNITED STATES OF AMERICA

## Abstract

*Chlamydia* are obligate intracellular bacterial pathogens that infect a wide range of vertebrate hosts. Despite having highly conserved genomes, closely related *Chlamydia* species can exhibit distinct host and tissue tropisms. The host tropisms of the human pathogen *Chlamydia trachomatis* and the closely related mouse pathogen *Chlamydia muridarum* are influenced by their ability to evade host immune responses, particularly those mediated by interferon gamma. However, there is evidence that tissue tropism is driven by additional poorly understood host and *Chlamydia* factors. In this study, we used a forward genetic approach to investigate the mechanisms that mediate *C. muridarum* tissue tropism. We conducted a tropism screen using a randomly mutagenized *C. muridarum* library and murine cell lines representing different tissues. We identified a mutant isolate whose growth was restricted in murine rectal and oviduct epithelial cells in an interferon gamma-independent manner. This phenotype was mapped to a missense mutation in *tc0237*, a gene that mediates the affinity of *C. muridarum* for cultured human epithelial cells. Our analysis of growth dynamics showed that the *tc0237* mutant exhibits a developmental delay in rectal epithelial cells. Together, these results suggest that TC0237 plays a role in *C. muridarum* tissue tropism.

## Introduction

Eukaryotic organisms have evolved diverse repertoires of innate immune defense mechanisms to detect and counter intracellular pathogens [1–3]. The ability of individual host cells to defend themselves, in the presence or absence of exogenous innate and adaptive immune signals, is known as cell-autonomous immunity (CAI) [4]. Conversely, the ability of intracellular pathogens to evade or subvert CAI in target cells of their definitive host(s) is essential for their survival [3].

*Chlamydia* is a genus of Gram-negative, obligate intracellular bacteria. These pathogens have a characteristic biphasic developmental cycle in which they alternate between two distinct cell forms: the infectious elementary body (EB) and the vegetative reticulate body (RB) [5,6]. Although most *Chlamydia* species (spp.) have narrow tissue and host tropisms in nature, their core genome is small and highly conserved [7]. Comparative genomic studies have revealed that the predictable nutrient environments within vertebrate cells have driven the decay of many of the corresponding biosynthetic pathways in *Chlamydia* genomes [8]. Conserved core genes are often sufficient for *Chlamydia* spp. to infect and proliferate in a broad range of vertebrate cell types *in vitro*, including cells from non-natural hosts [9]. In comparison, a smaller number of species- and strain-specific polymorphic and accessory effector genes dictate *Chlamydia* tissue and host tropism. Key targets of these effectors include CAI mechanisms that have diverged more than core metabolic processes in vertebrates [4].

The influence of CAI on *Chlamydia* tissue and host tropism is best understood for the human pathogen *C. trachomatis* (*Ctr*) and the closely related mouse pathogen *C. muridarum* (*Cmu*) [4,9]. Both pathogens productively infect a wide array of types of cultured mouse and human cells in the absence of interferon gamma (IFNγ), a key Th1 cytokine. However, IFNγ priming activates species-specific CAI mechanisms that restrict *Ctr* in mouse cells and *Cmu* in human cells [10–12]. Conserved and species-specific chlamydial effectors help these pathogens evade relevant CAI mechanisms in their definitive hosts. For example, *Ctr* strains use tryptophan synthase to produce tryptophan inside the chlamydial parasitophorous vacuole (inclusion), thereby circumventing the IFNγ-induced depletion of host cytosolic tryptophan by indoleamine 2,3-dioxygenase [10,13,14]. In mouse cells, the *Cmu* inclusion membrane protein GarD confers immunity to interferon-stimulated gene products and downstream effectors which are absent in human cells [15]. However, additional IFNγ-mediated and independent CAI mechanisms and corresponding chlamydial effectors contribute to tropism in cell culture and animal models [4,9].

Efforts to understand the roles of known IFNγ-mediated CAI mechanisms and cognate chlamydial effectors have been hampered by limitations of the animal models and genetic tools available for some *Chlamydia* [16]. Moreover, a variety of CAI-independent mechanisms, including differences in putative EB adhesins and distributions of cognate host ligands, are implicated in *Ctr* tissue tropism [13,17–24]. Collectively, prior observations highlight that chlamydial tropism is a complex phenotype, shaped by dynamic interplay between diverse host and pathogen factors.

## Methods

### Mammalian cell culture, *Chlamydia* infections, and Microscopy

Murine fibroblast (McCoy cells) and murine rectal carcinoma (CMT93) cell lines were obtained from the American Type Culture Collection (ATCC) and were maintained in high glucose Dulbecco’s Modified Eagle medium (DMEM; Cytiva Hyclone) supplemented with 10% fetal bovine serum (FBS; Atlanta Biologicals) non-essential amino acids, and HEPES (DMEM-10). Murine oviduct epithelial cells (C57) were a kind gift from Dr. Wilbert Derbigny and were cultured in 1:1 DMEM and F12K media (Sigma), supplemented with 10% FBS (Atlanta biologicals), 2 mM L-alanyl-L-glutamine, (Glutamax I; Gibco/Invitrogen), 5 µg/ml of bovine insulin, and 12.5ng/ml of recombinant human keratinocyte growth factor (Sigma) (DMEM/F12-10) as described [25]. All cell lines were maintained in humidified incubators at 37°C + 5% CO_2_ except when stated otherwise *Cmu* strain MoPn (*Cmu*^*wt*^) was a kind gift from Harlan D. Caldwell. *Cmu* strains were routinely cultured in McCoy cells. EBs were purified using a 30% MD-76R (Mallinckrodt Pharmaceuticals) cushion as described [26]. For routine infections, EBs or crude infection lysates were suspended in a cold sucrose-phosphate-glutamic acid (SPG) buffer and were added to confluent cell monolayers in cell-culture grade flasks or plates. Infections were performed using centrifugation-assisted or rocking infection protocols. For centrifugation-assisted infections, host cell monolayers were overlaid with pre-warmed culture medium, inoculated with an EB-SPG suspension, and centrifuged at 1600 RCF for 30 min at room temp. For rocking infections, the monolayers were overlaid with cold EBs-SPG and then were rocked at 37°C for 90 min. The SPG was aspirated and culture medium was added.

IFUs were determined at 24 hours post infection (hpi), unless stated otherwise. To fix the monolayers, the medium from infected monolayers was aspirated and 100% ice-cold methanol was added. Seven minutes later, the methanol was aspirated, and the monolayers were washed three times with phosphate buffered saline (PBS). Supernatant from a mouse hybridoma that produces an anti-chlamydia LPS antibody (EVI-HI) diluted 1:10 in PBS was used to label inclusions. Fixed monolayers were incubated with the supernatant for 1 h at room temp and then were washed three times with PBS. The monolayers were then incubated in the dark with an Alexa Fluor goat anti-mouse IgG 488-conjugated antibody (BioLegend, clone Poly4053) diluted 1:1000 in PBS. The monolayers were washed three times in PBS and inclusions were observed and counted using a Biotek Cytation 5 imaging multimode plate reader (Agilent) at 4X magnification. Inclusion counts, size, and cross-sectional areas were determined using onboard imaging software (Gen5v3.04).

### Mutant library construction

The temperature-sensitive *Cmu* strains CM^TS1^ and CM^*tsp*^ were previously isolated from a heavily mutagenized *Cmu* library [27,28]. Here, we used CM^TS1^ to generate a new *Cmu* mutant library similarly as we described previously, except that the infected McCoy cell cultures were exposed to 1.5 µg/ml ethyl methanesulfonate (EMS) for 60 min [27].

### Tropism screen

McCoy, CMT93, and C57 cell lines were seeded in 96-well plates 48 h prior to screening. Twenty-four h later, the medium was replaced with 100 µl/well fresh culture medium + / − 20 units/ml of recombinant mouse IFNγ (R&D Systems), an overview of the screen is shown in supplementary Fig 1 (S1 Fig). Library isolates were thawed at room temperature for 30 min and then were diluted 1:10 in SPG. EBs-SPG were used to inoculate parallel 96-well plates as technical singlets. *Cmu*^*wt*^, CM^TS1^, and Igs4 (a previously characterized IFNγ-sensitive *Cmu* mutant [27]) were included on each screen plate as controls. The cells were infected by centrifugation at 1400 RCF for 30 min at room temp. Fresh medium + / − IFNγ was added, and then the infections were incubated for 24 h. At 24 hpi, the infected cells were fixed with methanol, inclusions were labeled with antibodies, and IFU were determined as above. The images were also manually screened to identify abnormal inclusions that could not be accurately counted using the imaging software.

Library isolates that had IFU ratios below 2 standard deviations of CM^TS1^ were flagged as potential tropism mutants. Library isolates that grew poorly in all conditions or grew similarly to the CM^TS1^ parent were eliminated from further analysis. The secondary screen was performed identically to the primary screen, but in quadruplicate. Finally, M7 and M8 were plaque purified twice and expanded in McCoy cells.

### Whole genome sequencing, assembly, and analysis

Crude EBs harvested from McCoy cells were pelleted by centrifugation and the pellet was treated with DNase I (Promega) to remove residual host DNA and DNA from lysed EBs. Whole genome amplification was performed using the REPLI-g mini kit (Qiagen) as described [29]. DNA was quantified with the Quant-It dsDNA High-Sensitivity Assay kit (Life Technologies). Sequencing libraries were prepared using the Nextera XT DNA Library Preparation Kit (Illumina) and multiplexed as described [29]. Paired-end reads were sequenced on a NovaSeq X Plus device in the Center for Medical Genomics at Indiana University School of Medicine.

Mutations (SNPs and nucleotide insertions/deletions [Indel]) were identified in the raw sequence data by comparison to a *Cmu* reference genome (AE002160.2) as described [28]. Putative mutations were confirmed by PCR and Sanger sequencing (Eurofins Genomics) (S1 Table).

### Mapping mutant alleles

McCoy cell monolayers were co-infected with library isolates and CM^*tsp*^ (MOI of 2 per mutant) using centrifugation as described [28]. Co-infections were incubated at 37°C for 36–40 hours (two developmental cycles). The medium was then aspirated, SPG and glass beads were added, and the infected cells were detached and lysed by bead agitation. The lysates were used to infect fresh McCoy cells, and the infections were incubated at 40°C for 24 hours. The infection and blind passage steps were repeated two more times at 40°C to ensure elimination of the temperature sensitive parents. Surviving temperature resistant recombinants were then plaque isolated, expanded in McCoy cells, and phenotyped. The *tco237* genotypes of the recombinants were determined by PCR and Sanger sequencing.

### Construction of pTC0237-FLAG

Purified shuttle vector p2TK2Nigg*Spec*^*R*^mCherryTetR::*tc0273*:3xFLAG was used as the template to PCR amplify the vector backbone (NEB Q5 Polymerase; Cat. No. #M0491) (S1 Table) [30]. PCR reactions were pooled and treated with DpnI (NEB, Cat. No. R0176) according to the manufacturer instructions. The linearized vector was mixed with Q5 polymerase and a PCR amplicon of *tc0237*^*wt*^ in which ~20 base pair homologous 5’ and 3’ ends were introduced in the primers (S1 Table) at a molar ratio of 1:5. Assembly was performed at 50°C for two hours using NEBuilder HiFi DNA assembly master mix (Cat. No. E2621). The reaction product was transformed into *E. coli,* and the cells were plated on Luria-Bertani agar plates supplemented with spectinomycin and incubated at 30°C. The final expression vector, pTC0237-FLAG was confirmed by whole plasmid sequencing (Eurofins Genomics). C*mu* was transformed with pTC0237-FLAG as described [31]. The transformants were expanded in McCoy monolayers in DMEM-10 supplemented with 1 µg/ml cycloheximide (CHX), 100 µg/ml spectinomycin, and either 20 ng/ml anhydrotetracycline (aTc) in 100% dimethyl sulfoxide (DMSO) or an equal volume of 100% DMSO. EBs were purified as above.

### Two-step RT-qPCR

McCoy cells were infected with EBs-SPG at an MOI of 0.5 by rocking. The infected cells were lysed at various hpi with equal volumes of TRIzol (100 µl/cm^2^) and sterile glass beads. Total RNA was purified using the Direct-Zol RNA Miniprep kit (Zymo Research, Cat. No. R2052). The purified RNA was treated twice with DNase I and was divided into aliquots. The RNA was reverse transcribed using Maxima H minus reverse transcriptase master mix (Thermo Scientific, Cat. No. M1661) and random hexamer primers to generate cDNA, or without RNA template to generate no-reverse-transcriptase controls. The RT reaction products were diluted 1:5 in molecular-grade water and used as templates in quantitative PCR (qPCR). SnapGene (v7.2.1) was used to design primers for *Cmu* 16s rRNA, *tc0237*, and *tc0237*:3xFLAG transcripts (S1 Table). The qPCR reactions were performed using PowerUp SYBR Green Master Mix (Thermo Scientific, Cat. No. A25742) on an Azure Cielo Real-time PCR System (Azure Biosystems). Known concentrations of PCR-amplified targets were used to generate standard curves.

### Progeny assay and one-step growth curves

McCoy and CMT93 cells grown in 12 or 24-well plates were infected with EBs-SPG at an MOI of 0.1 or were mock-infected with SPG alone using rocking or centrifugation. IFUs were determined in McCoy cells in 96-well plates using centrifugation. EBs were harvested at various hpi using bead agitation. Progeny IFU was compared to input IFU to calculate burst size.

### Statistical analysis

Statistical analyses were conducted using GraphPad Prism (v10.4.1). The specific tests used are indicated in the figure legends.

## Results

### A forward genetic screen identifies a gene that mediates *Cmu* epithelial tropism

To search for *Cmu* mutants with IFNγ-independent tropism defects, we compared the number of inclusion forming units (IFU) that isolates from a new mutant library formed in murine fibroblast and epithelial cell lines in the presence and absence of IFNγ (+/ − IFNγ). We constructed the mutant library from CM^TS1^, a temperature sensitive mutant that develops normally at 37°C but fails to form infectious EBs at 40°C [27,28]. The rationale was that this would allow us to use lateral gene transfer to map mutant alleles by performing co-infections with another temperature sensitive mutant, CM^*tsp*^ and isolating temperature resistant recombinants, similarly as we described [29,32]. We mutagenized CM^TS1^ by supplementing the cell culture medium of infected mouse McCoy fibroblasts with a low dose of the transition-inducing mutagen EMS [33]. We then plaque-cloned 4,932 isolates from the infection lysate and expanded the isolates in McCoy cells treated with the eukaryotic translation inhibitor CHX.

In a primary screen performed in singlet, we compared the IFUs that equal inocula of each library isolate formed in McCoy, oviduct epithelial C57 [25] and in CMT93 rectal epithelial cells [34] +/ − IFNγ, at 24 hpi (S1 Fig). Using the IFU ratio that eight replicates of CM^TS1^ formed in McCoy verses CMT93 cells and in McCoy verses C57 cells as references, we identified library isolates whose IFU ratios differed by less than two standard deviations. We then excluded isolates that formed few inclusions and or grew slowly in McCoy cells + / − IFNγ. Finally, we repeated the screen in quadruplicate with the remaining isolates.

**Fig 1:**
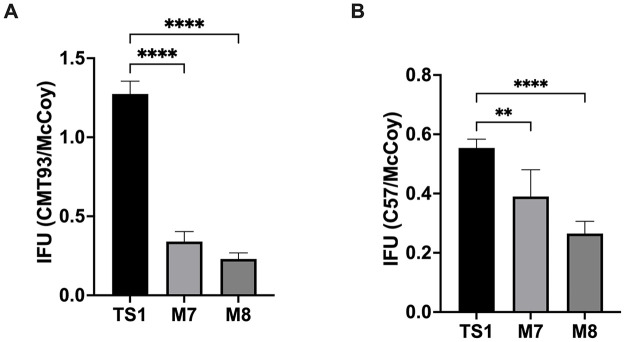
M7 and M8 IFUs are reduced in CMT93 and C57 compared to McCoy cells in the absence of IFNγ. Equal inoculums of CM^TS1^, M7, or M8 were used to infect CMT93, C57, and McCoy cells, and IFU were counted at 24 hpi. The ratio of IFUs that the strains formed in **(A)** CMT93 versus McCoy cells and in **(B)** C57 versus McCoy cells were compared. Significance was determined by ordinary one-way ANOVA with Dunnett’s multiple comparisons posttest. Error bars represent standard deviation. **, P < 0.01; ****, P < 0.0001.

**Fig 2:**
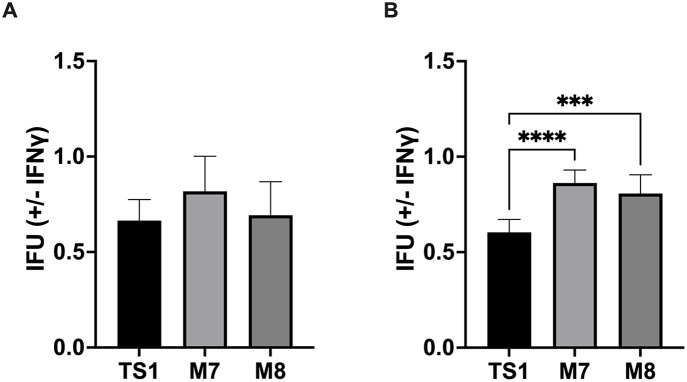
M7 and M8 are as resistant to IFNγ as CM^TS1^ in epithelial cells. Equal inocula of CM^TS1^, M7, or M8 were used to infect **(A)** CMT93 and **(B)** C57 cells + / − IFNγ, and IFU were counted at 24 hpi. IFUs that the strains formed in the cell lines + / − IFNγ were compared. Significance was determined by ordinary one-way ANOVA with Dunnett’s multiple comparison posttest. Error bars represent standard deviation. ***, P < 0.001; ****, P < 0.0001.

We identified two library isolates, M7 and M8, in the secondary screen that consistently exhibited reduced inclusion formation in CMT93 and C57 cells. In the absence of IFNγ priming, M7 and M8 IFUs were reduced, on average, 66% and 77% and 61% and 73% in CMT93 and C57 compared to McCoy cells, respectively (Fig 1A–B). In contrast, compared to CM^TS1^, M7 and M8 had similar IFU ratios + / − IFNγ in CMT93 cells and produced higher IFU ratios + / − IFNγ in C57 cells (Fig 2). These observations suggested that M7 and M8 have interferon-independent epithelial-specific growth defects.

### Restricted phenotypes map to a mutant *tc0237* allele

We sequenced the genomes of CM^TS1^, M7 and M8 to search for potential EMS-induced single nucleotide polymorphisms (SNPs). The genomes of the mutants were identical, suggesting that they are sibs. The mutants only differed from CM^TS1^ by a single SNP in *tc0237*^T→G^ (position 179, *tc0237*^*mut*^) predicted to cause an asparagine to threonine change in TC0237 (N60T). To determine if *tc0237*^*mut*^ segregated with the mutant phenotypes, McCoy cells were co-infected with M8 and CM^*tsp*^ at 37°C, the resulting lysates were used to infect McCoy cells, and then the infections were incubated at 40°C. After two more blind passages in McCoy cells at 40°C, we plaque isolated twenty-four temperature resistant recombinants from the infection lysates and determined their *tc0237* genotypes using PCR amplification and Sanger sequencing. We selected three recombinants with *tc0237*^*mut*^ (M8R4, M8R5, M8R15) and three with *tc0237*^*wt*^ (M8R21, M8R23, M8R24) for further characterization.

**Fig 3:**
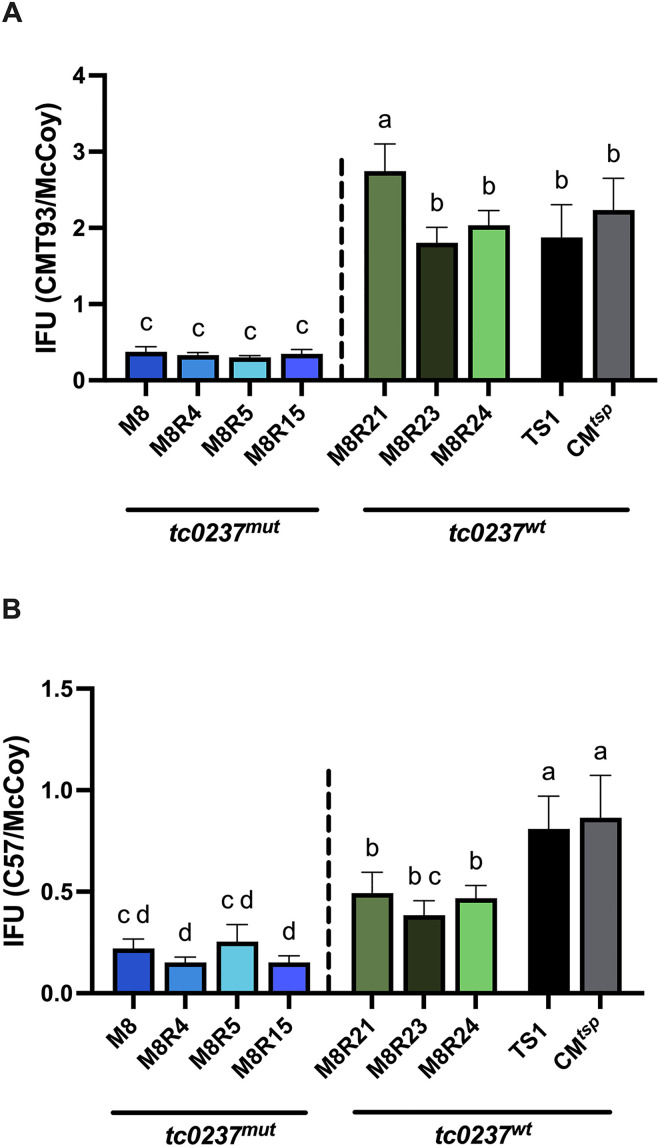
*tc0237* mediates *Cmu* epithelial cell tropism. M8, various recombinants, CM^TS1^, or CM^*tsp*^ were used to infect McCoy, (**A**) CMT93, and (**B**) C57 cells at MOIs of 1.0, and inclusions were counted at 24 hpi. Ratios of IFUs that the strains formed in CMT93 or C57 cells divided by the IFUs formed in McCoy cells are shown on the y-axis. Significance was determined by ordinary one-way ANOVA with Tukey’s multiple comparison test. Shared letters indicate groups with p-values greater than 0.05. Groups that do not share a letter have p-values less than 0.05. Error bars represent standard deviation.

We compared IFU production of the recombinants and the parents in C57, CMT93, and McCoy cells. In CMT93 cells, reduced IFUs perfectly segregated with *tc0237*^*mut*^ (Fig 3A). M8 and all recombinants with *tc0237*^*mut*^ had similarly reduced IFU ratios in CMT93 versus McCoy cells compared to CM^TS1^ and CM^*tsp*^, and *tc0237*^*wt*^ recombinants_._ The effect of *tc0237*^*mut*^ was more nuanced in C57 cells (Fig 3B). Additionally, while M8 *tc0237*^*wt*^ recombinants had higher IFU ratios in C57 versus McCoy cells compared to the *tc0237*^*mut*^ recombinants, the *tc0237*^*wt*^ recombinants had lower IFU ratios compared to CM^TS1^ and CM^*tsp*^. These observations confirmed that *tc0237* plays a role in *Cmu* epithelial cell tropism *in vitro* and suggested that this role could be more important in rectal compared to oviduct epithelial cells.

To determine if *tc0237*^*mut*^ acts *in cis or trans,* we PCR amplified *tc0237*^*mut*^ from CM^TS1^ and cloned it into the shuttle vector p2TK2Nigg*Spec*^*R*^mCherryTetR to generate p2TK2Nigg*Spec*^*R*^mCherryTetR::*tc0237*^*wt*^:3xFLAG (pTC0237-FLAG) [22]. We then transformed this expression vector into M8R4^*237mut*^ to create the complement strain M8R4-pTC0237-FLAG. In contrast, several attempts to transform M8R4^*237mut*^ with the empty shuttle vector failed for unknown reasons. We confirmed that M8R4-pTC0237-FLAG expressed *tc0237*-FLAG transcript in both the absence and presence of aTc, and that aTc increased the number of these transcripts, but we were unable confirm expression of TC0237-FLAG protein by Western blot with anti-FLAG antibodies (Sigma Aldrich; clone M2 and rabbit polyclonal F7425), possibly due to low levels of expression or because TC0237 is unstable (Fig S2).

**Fig 4:**
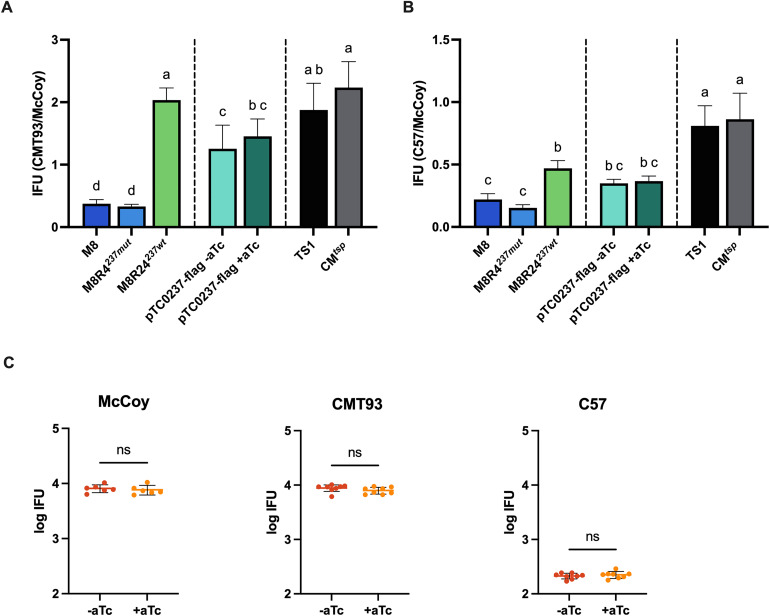
Expression of TC0237-FLAG *in trans* increases M8R4^237*mut*^ IFU formation in epithelial. McCoy cells were infected with M8R4-pTC0237-FLAG at an MOI of 1.0 in +/ − aTc and the infected cells were lysed 24 hours later. The lysates, M8, M8R4^237*mut*^, M8R24^237*wt*^, CM^TS1^, and CM^*tsp*^ were used to infect fresh McCoy, **(A)** CMT93, or **(B)** C57 cells, and IFUs were determined at 24 hpi. **(C)** McCoy, CMT93, or C57 cells were infected with M8R4-pTC0237-FLAG at MOIs of 0.1, fresh medium + / − aTc was added at 2 hpi, and IFUs were determined at 24 hpi. The number of IFUs formed in each condition is on the y-axis and each dot represents the result of a technical replicate. Significance was determined by (**A-B**) ordinary one-way ANOVA with Tukey’s multiple comparison test or (**C**) unpaired t-test. Shared letters indicate groups with p-values greater than 0.05. Groups that do not share a letter have p-values less than 0.05. Error bars represent standard deviation. ns = not significant.

We used two approaches to assess if TC0237-FLAG increased IFUs of M8R4^237*mut*^ in CMT93 and C57 epithelial cells (Fig 4). First, McCoy cells were infected with M8R4-pTC0237-FLAG and the infections were incubated in cell culture medium + / − 20 ng/ml aTc, and then the infected cells were lysed at 24 hpi. These lysates or M8, M8R4^*237mut*^, M8R24^237*wt*^, CM^TS1^, and CM^*tsp*^ were then used to infect CMT93, C57, and McCoy cells, and IFUs were determined at 24 hpi. M8R4-pTC0237-FLAG + / − aTc produced more IFUs in CMT93 versus McCoy compared to M8 and M8R4^*237mut*^, but reduced IFUs compared to M8R24^237*wt*^, CM^TS1^, and CM^*tsp*^ (Fig 4A). This suggested that pTC0237-FLAG was sufficient to partially restore infection of CMT93 cells, and that leaky expression from pTC0237-FLAG was sufficient to mediate this. M8R4-pTC0237-FLAG + / − aTc produced a slightly higher IFU ratio in C57 versus McCoy cells compared to M8 and M8R4^*237mut*^, and a slightly reduced IFU ratio compared to M8R24^237*wt*^, but these differences were not significant (Fig 4B). M8R4-pTC0237-FLAG + / − aTc, M8, M8R4^*237mut*^, M8R24^237*wt*^, produced lower IFU ratios in C57 versus McCoy cells when compared to CM^TS1^ and CM^*tsp*^, further suggesting that *tc0237*^*wt*^ could play a more important role in rectal epithelial cells than in oviduct epithelial cells. We additionally tested if adding aTc to the cell culture medium at 2 hpi impacted M8R4-pTC0237-FLAG IFUs in McCoy, CMT93, and C57 cells but did not observe any differences (Fig 4C).

### *tc0237* mutants have altered growth dynamics in rectal epithelia cells

**Fig 5:**
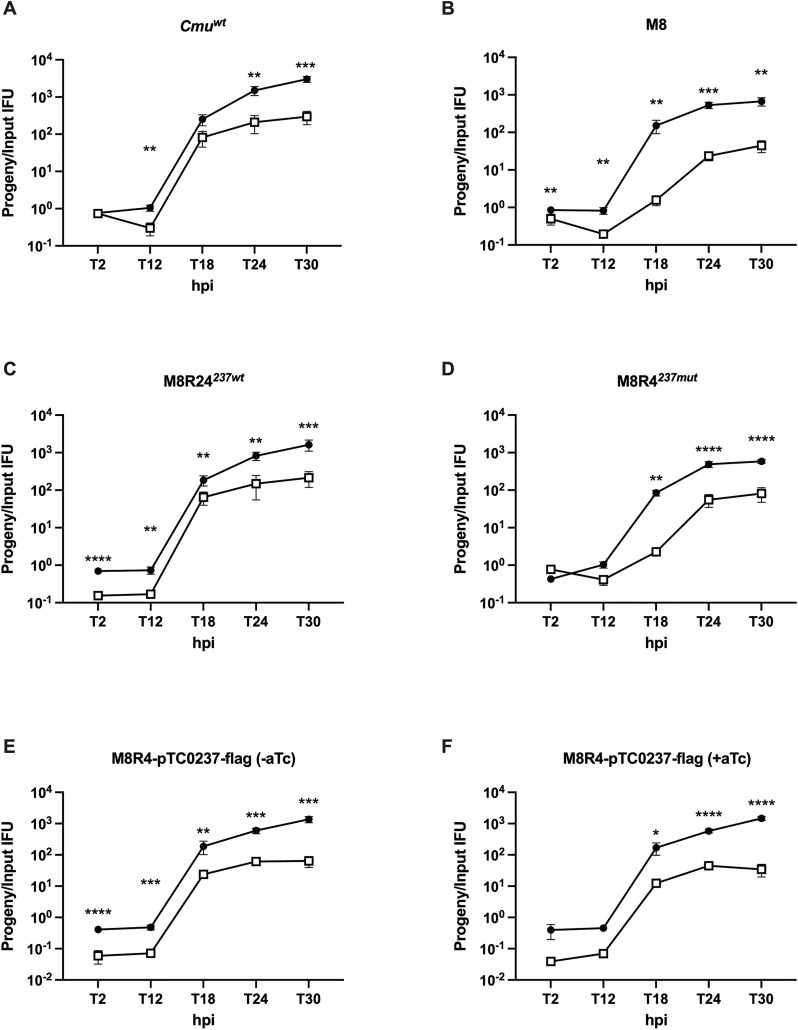
*tc0237* mutants have altered growth dynamics in CMT93 cells in centrifugation-assisted conditions. McCoy (black circles) and CMT93 (white squares) cells were infected with *Cmu*^*wt*^, M8, M8R4^*237mut*^, M8R24^*237wt*^, and M8R4-pTC237-FLAG + / − aTc. The number of IFUs produced by these infections was compared to the input inoculum of the primary infection to determine the relative burst size (y-axis) at various hpi (x-axis). Relative burst sizes of infections in McCoy and CMT93 cells were compared by ordinary two-way ANOVA with Bonferroni correction. Graph shows the results from two independent trials, in technical triplicates. Error bars represent standard deviation. *, p < 0.05; **, p < 0.01; ***, p < 0.001; ****, p < 0.0001.

**Fig 6:**
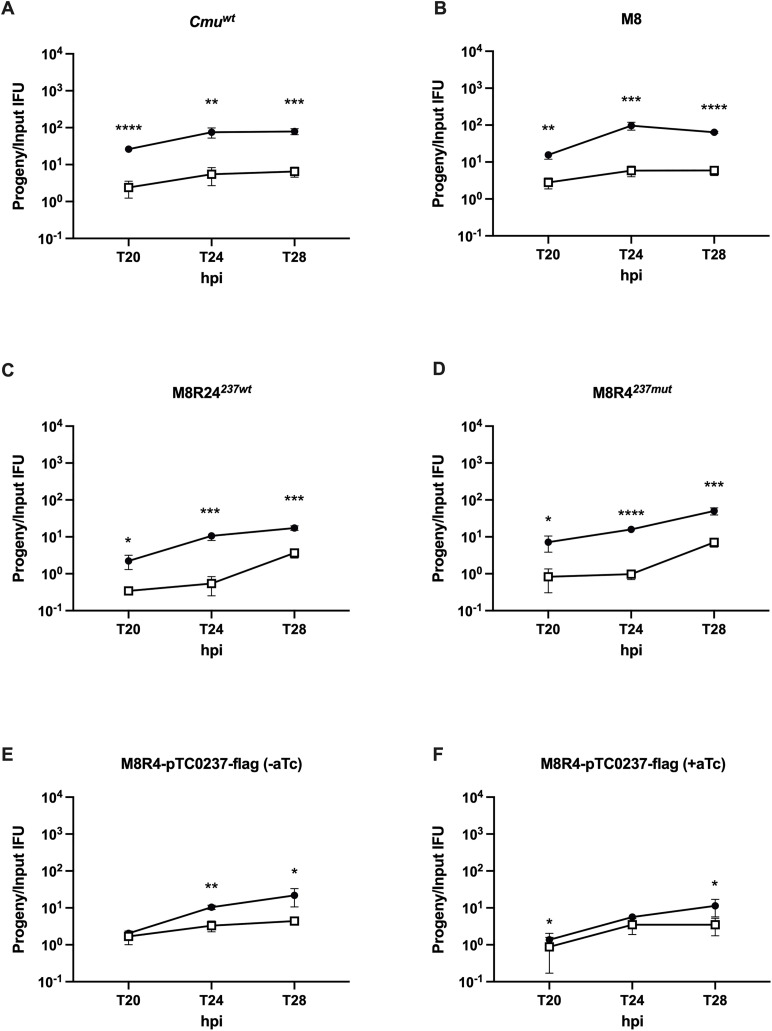
*tc0237* alleles do not alter attachment affinity to McCoy or CMT93 cells. McCoy (black circles) and CMT93 (white squares) cells were infected with *Cmu*^*wt*^, M8, M8R4^*237mut*^, M8R24^*237wt*^, and M8R4-pTC237-FLAG + / − aTc without centrifugation. The number of IFUs produced was compared to the input IFUs of the primary infection to determine the relative burst size (y-axis) at various hpi (x-axis). Relative burst sizes of infections in McCoy and CMT93 cells were compared by ordinary two-way ANOVA with Bonferroni correction. The graphs show the results from two independent experiment performed in technical triplicates. Error bars indicate standard deviation. *, p < 0.05; **, p < 0.01; ***, p < 0.001; ****, p < 0.0001.

We performed one-step growth curves to attempt to determine why M8 formed fewer IFUs in epithelial cells. McCoy and CMT93 cells were infected with various strains at MOIs of 0.1 as in prior experiments (Fig 5). The infected host cells were lysed at different hpi, and IFUs were determined in McCoy cells. By 18 hpi, IFU production of the wild type CM^TS1^ parent (*Cmu*^*wt*^) had nearly peaked in both cell lines. Although M8 produced similar IFUs in McCoy cells at 18 hpi compared to *Cmu*^*wt*^, it produced far fewer IFUs in CMT93 cells compared to *Cmu*^*wt*^. Consistent with the possibility that this was due to the *tc0237*^*mut*^, IFU production of M8R4^*237mut*^ at 18 hpi in CMT93 cells was similar to M8, whereas IFU production of M8R24^*237wt*^ was similar to *Cmu*^*wt*^. IFU production of M8R4-pTC0237-FLAG + / − aTc in CMT93 cells at 18 hpi was also more similar to *Cmu*^*wt*^. Another group reported that attachment of a different *Cmu tc0237* mutant to HeLa cells was increased in cell culture relative to the parent [35], and since attachment of some *Chlamydia* spp. in cell culture is altered by centrifugation [36], we repeated the one-step growth curve analysis without centrifugation (Fig 6). Relative burst sizes of all of the strains were reduced, and there was no obvious relationship between this phenotype and *tc0237* genotype. Collectively, these results suggested that the *tc0237*^*mut*^ causes a developmental delay during centrifugation-assisted infection of CMT93 cells.

## Discussion

In *Cmu*, *tc0237* is encoded in an operon with two paralogs, *tc0236* and *tc0235.* All three genes encode DUF 720 domain protein-coding genes. Nucleotide BLAST identified highly conserved orthologs in other *Chlamydia* spp. *Cmu tc0237* shares >90% homology with *Ctr* and *C. suis* orthologs and >50% homology with orthologs in more distantly related *Chlamydia* spp*. Ctr tc0237–235* orthologs were secreted by a *Yersinia* spp. surrogate type III secretion system (T3SS), suggesting that these genes are T3SS-secreted chlamydial effectors proteins [37,38]. TC0237 is predicted to contain a coiled-coil structural motif (residues 81–115) nested within the DUF 720 region (residues 31–154), that may permit the alpha helices to interact with one another [39]. Unfortunately, we have not been able to confirm this interaction using independent approaches because our attempts to generate high affinity antibodies to TC0237 failed. We are attempting to generate new antibodies and fusion constructs to improve TC0237 detection. Localization of TC0237 in *Cmu* and host cells and identification of potential interaction partners are key future directions.

Several observations from our and prior studies suggest that TC0237 plays a role in chlamydial tropism. Chen and colleagues identified a *Cmu* strain with mutations in *tc0237* and *tc0668* using Pasteurian selection in HeLa cells and subsequently isolated clones that segregated these mutant alleles [35,40,41]. The clones with mutant *tc0237* formed more inclusions than the parent in HeLa cells during rocking infection, similar to the infection approach they used during Pasteurian selection, but not during centrifugation-assisted infection. This led them to conclude that TC0237 plays an unspecified role in EB affinity for target cells. The double mutant was severely attenuated in a mouse genital tract infection model, but the *tc0237* mutant was not [35,40,42]. However, subsequent studies revealed the *tc0237* was attenuated in mouse gastrointestinal infection models [43]. Since *Cmu* is spread fecal-orally, and not sexually, in rodents in nature [44], these results suggest that TC0237 is most relevant in its natural niche in its definitive host. Here, we searched for *Cmu* mutants that formed fewer inclusions in more relevant mouse epithelial cells compared to less relevant mouse fibroblasts, with the caveat that our approach still represents a limited surrogate model. In contrast to the observation of Chen and colleagues in HeLa cells, the ability of our *Cmu*^*237mut*^ isolates to infect relevant mouse epithelial cells was attenuated in centrifugation-assisted compared to rocking infection [35]. Phenotyping our mutants in parallel with the mutants identified by Chen and colleagues will be a critical next step to determine if they have similar loss of function mutations.

We think that prior observations and our findings are complementary and suggest an exciting tropism-related function for TC0237, contingent upon the hypothesis that *Cmu* employs redundant attachment/entry mechanisms in some cells. We speculate that TC0237 interacts with a host ligand to regulate attachment/entry into preferred host cell types and blocks infection of cells that lack this ligand. Presumably, TC0237 blocks infection of non-native HeLa cells during rocking conditions because these cells do not display the ligand, but this is circumvented by alternate attachment/entry mechanisms engaged by centrifugation. In contrast, TC0237 may be dispensable for infection during rocking infection because the relative burst sizes of *Cmu*^*wt*^ and *Cmu*^*237mut*^ were low and similar in the mouse cell lines we tested. In contrast, we speculate that loss of TC0237 is detrimental during centrifugation-assisted infection of mouse epithelial cells because TC0237-ligand interactions help *Cmu* circumvent an epithelial-cell specific CAI mechanism that is engaged by centrifugation.

## Supporting information

S1 Fig Schematic overview of the tropism screen. Equal inoculums of the library mutants were used to infect parallel plates of the indicated cells in the presence or absence of IFNγ and CHX as indicated and then inclusions were counted at 24 hpi.
(PNG)

S2 Fig Detection of recombinant pTC0237-FLAG transcripts in McCoy cells. M8R4-pTC0237-FLAG infected McCoy cells were lysed at the indicated hpi, and DNA-free RNA was isolated. TC0237-FLAG transcripts were measured using qRT-PCR and were quantified by comparison to a standard curve of pTC0237-FLAG concentrations. The graph shows the averages from technical triplicates, and the error bars indicate standard deviation. Significance was determined by two-way ANOVA with Bonferroni’s posttest corrections. *, P < 0.05; **, P < 0.01.
(TIFF)

S1 Table List of primers used in this study. (DOCX)
