# Supplementary figures and images for: Isolation and characterization of a Chlamydia muridarum tc0237 mutant from a genetic screen that is attenuated in epithelial cells
Source: PLoS One. 2025 Aug 5;20(8):e0329637. doi: 10.1371/journal.pone.0329637 (PMC12324114; doi:10.1371/journal.pone.0329637)

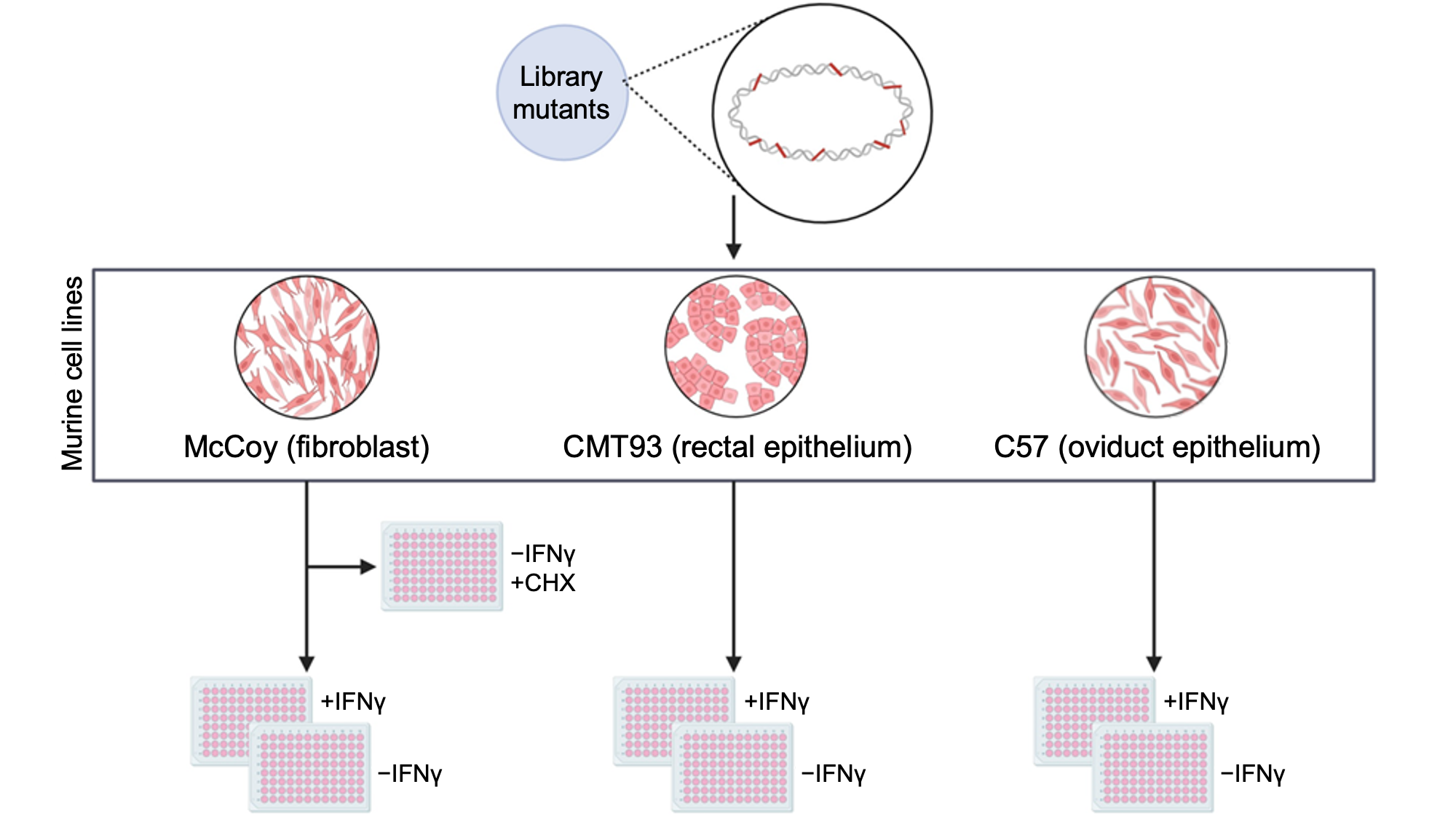

Supplement: S1 Fig — Equal inoculums of the library mutants were used to infect parallel plates of the indicated cells in the presence or absence of IFNγ and CHX as indicated and then inclusions were counted at 24 hpi. (PNG) [file pone.0329637.s001.png]

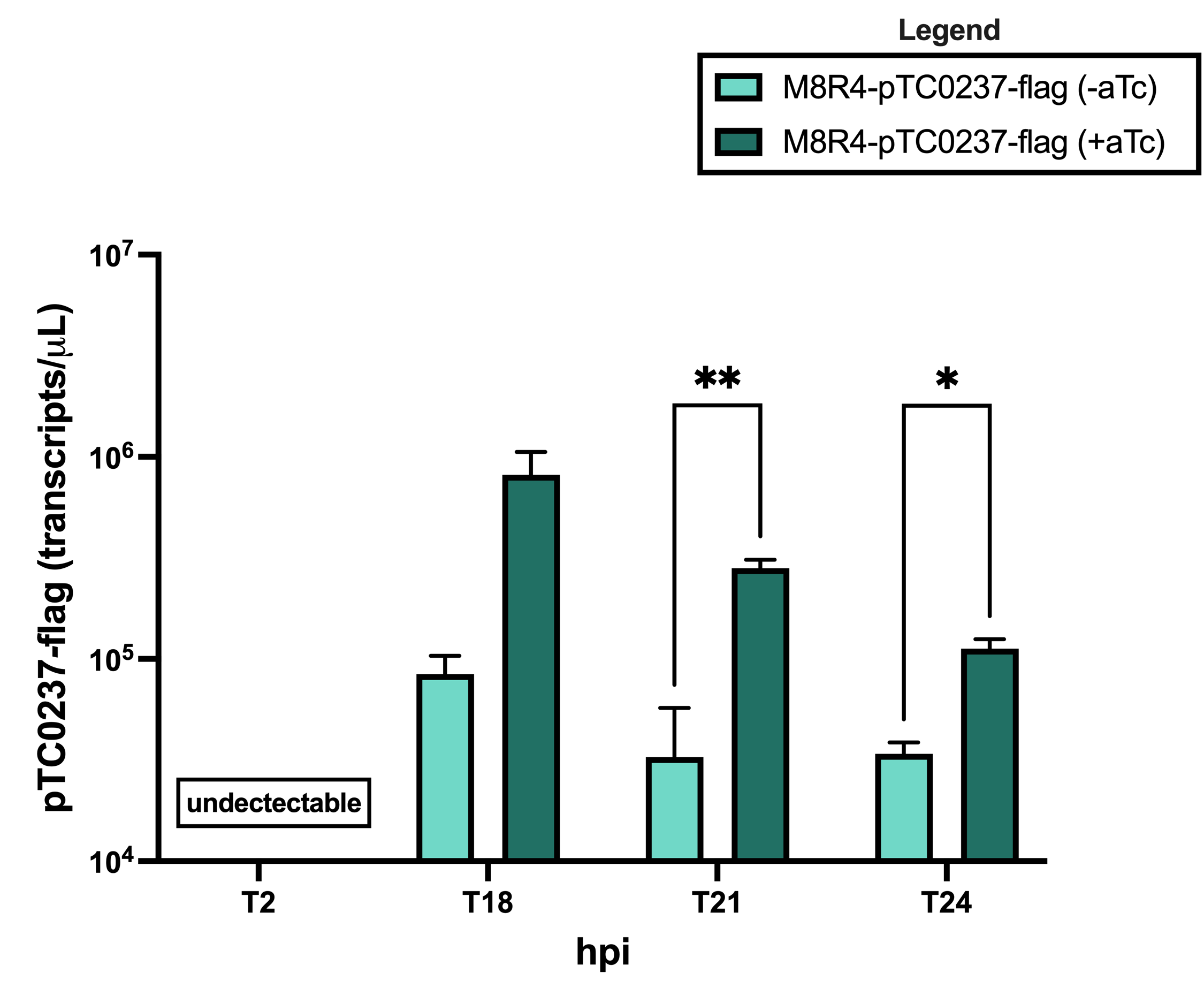

Supplement: S2 Fig — M8R4-pTC0237-FLAG infected McCoy cells were lysed at the indicated hpi, and DNA-free RNA was isolated. TC0237-FLAG transcripts were measured using qRT-PCR and were quantified by comparison to a standard curve of pTC0237-FLAG concentrations. The graph shows the averages from technical triplicates, and the error bars indicate standard deviation. Significance was determined by two-way ANOVA with Bonferroni’s posttest corrections. *, P < 0.05; **, P < 0.01. (TIFF) [file pone.0329637.s002.tiff]
