# Supplementary material for: Isolation and characterization of a Chlamydia muridarum tc0237 mutant from a genetic screen that is attenuated in epithelial cells
Source: PLoS One. 2025 Aug 5;20(8):e0329637. doi: 10.1371/journal.pone.0329637 (PMC12324114; doi:10.1371/journal.pone.0329637)
Supplement: S1 Table — (DOCX) [file pone.0329637.s003.docx]

| **Oligo Name** | **Oligo Sequence (5' to 3')** | **Description** |
| --- | --- | --- |
| DN2670:TC0237-S | ATGTCAGCACCTATCCCACC | *tc0237* SNP confirmation |
| DN2671:TC0237-AS | TTAACTGTGCTACCTACGGAGTTC |  |
| DN2620:p2TK2CmTetFlag-S | ATGGACTACAAAGACCATGACG | Linearization of p2TK2NiggSpecRmCherryTetR::*tc0273:*3xFLAG |
| DN2621:p2TK2CmTetFlag-AS | TTCACTTTTCTCTATCACTGATAGG |  |
| DN2622:TC0237Comp-S | CTATCAGTGATAGAGAAAAGTGAAATGTCAGCACCTATCCCACCAGC | Amplification of *tc0237* with p2TK2Nigg*SpecR*mCherryTetR::  3xFLAG overlap |
| DN2623:TC0237Comp-AS | GTCATGGTCTTTGTAGTCCATCCCCCCCCCAGACAGGGGTTTGTTTAACTG |  |
| DN2636:p2TK2CmScrn-S | ATCTAGTGCTTACGACCATCC | Colony PCR screening primers |
| DN2637:p2TK2CmScrn-AS | CGTGTGATTTCGTCCTCTTTG |  |
| MoPn16SrRNA-S | TTTAGTGGCGGAAGGGTTAG | qPCR Primers |
| MoPn16SrRNA-AS | TCTACGCCACATTCGGTATTAG |  |
| DN2670:TC0237-S | ATGTCAGCACCTATCCCACC |  |
| DN2682:TC237RTQPCR-AS | TTAAGACAGGGGTTTGTTTAACTG |  |
| DN2684:3xFLAGTag-AS | TTAATCGTCATCCTTGTAATCGATGTC |  |

**S1 Table**: List of Primers used in this study.
